# Undernutrition in Infants Aged Under Six Months: A Multi-Center Cross-Sectional Study in Two Governorates in Yemen

**DOI:** 10.3390/nu18142283

**Published:** 2026-07-12

**Authors:** Maha Ahmed Basodan, Iman Ali Ba-Saddik, Michael Boele van Hensbroek, Wieger Voskuijl

**Affiliations:** 1 Center for Global Child Health, Emma’s Children Hospital, Amsterdam UMC, Location AMC, University of Amsterdam, 1105 AZ Amsterdam, The Netherlands; 2 Amsterdam Center for Global Health, Department of Pediatrics and Department of Global Health, Amsterdam UMC, 1105 AZ Amsterdam, The Netherlands; m.boele@amsterdamumc.nl (M.B.v.H.); w.p.voskuijl@amsterdamumc.nl (W.V.); 3 Faculty of Medicine and Health Sciences, University of Aden, Crater, Aden P.O. Box 878, Yemen; iman.ali.med@aden-uni.net; 4 Al-Wahda Teaching Hospital, Al-Shikh Othman, Aden P.O. Box 420, Yemen; 5 Department of Paediatrics and Child Health, Kamuzu University of Health Sciences, Blantyre P/Bag 360, Malawi

**Keywords:** anthropometric deficits, infants under 6 months, malnutrition, undernutrition, the composite index of anthropometric failure

## Abstract

**Background/Objectives**: Data on undernutrition among infants under six months (<6 m) in Yemen are limited. This study aimed to estimate the prevalence of conventional, concurrent and severe forms of anthropometric failure among infants < 6 m attending routine health visits in two Yemeni governorates and to determine the anthropometric indicator that shows greater overlap with these failures. **Methods**: This facility-based survey was conducted over thirteen months in five health facilities. We measured infants’ weight, length and mid-upper arm circumference (MUAC). Weight-for-age (WAZ), length-for-age (LAZ), and weight-for-length z-scores (WLZ), composite index of anthropometric failure (CIAF), and composite index of severe anthropometric failure (CISAF) were calculated. Additionally, we assessed the overlap of different MUAC thresholds and WAZ < −2 with various forms of anthropometric failure. **Results**: In total, 5053 infants were analyzed. Further, 19.3% (95% CI: 15.1; 24.3), 17.7% (95% CI: 13.8; 22.5), and 30.5% (95% CI: 21.8; 40.7) were wasted, stunted and underweight, respectively. Overall, 40.9% (95% CI: 34.7; 47.5) were classified as (CIAF). Within the CIAF group, 17.7% (95% CI: 12.5; 24.4) had a single anthropometric failure and 23.3% (95% CI: 17.9; 29.7) had concurrent failures. Furthermore, 12% (95% CI: 10.8; 13.4) of all infants exhibited the most severe forms of anthropometric failure (CISAF). WAZ < −2 overlapped with a significantly higher proportion of infants with anthropometric failure compared to MUAC: CIAF (74.4% vs. 28.8%) and CISAF (92.1% vs. 47%). **Conclusions**: Conventional, concurrent and severe anthropometric failures are common among <6 m infants in Yemen. WAZ captured more at-risk infants than MUAC.

## 1. Introduction

The first six months of life are important in terms of a child’s growth [1]. This period is characterized by rapid physical changes in which nutrition plays a key role [2], making it a period of high vulnerability to undernutrition [3,4].

Anthropometry is used to assess children’s nutritional status, including under-six-month (<6 m) infants. Using WHO growth standards, the individual forms of anthropometric failures, wasting, stunting, and underweight, are defined as Z-scores below −2 (severe < −3) for weight-for-length (WLZ), length-for-age (LAZ), and weight-for-age (WAZ), respectively [5]. These conventional anthropometric indicators do not consider that some infants may have more than one form of growth failure. Therefore, the composite index of anthropometric failure (CIAF) has been introduced as a comprehensive indicator to capture the total burden of anthropometric failure [6,7]. The CIAF includes three single forms of failures (wasted only, stunted only, and underweight only) and three concurrent forms (wasted and underweight; wasted, stunted and underweight; and stunted and underweight) [6]. The coexistence of multiple anthropometric failures, even if the individual failure is moderate, is associated with an increased risk of morbidity and mortality [8].

Multiple studies suggest that mid-upper arm circumference (MUAC) more reliably indicates <6 m infants’ anthropometric failure and mortality risk than length-based measures [9,10,11,12]; the MUAC cut offs range from 9.5 to 11.5 cm [13,14,15]. Recently, the World Health Organization (WHO) recognized MUAC < 11 cm to identify infants aged 6 weeks to 6 months at risk of poor growth and development [16]. One issue remains debated for infants < 6 m: while multiple studies show MUAC and WAZ outperform WLZ in predicting mortality and growth failure [15,17,18], there is no consensus which indicator best captures different forms of anthropometric failure in this age group.

Yemen is the poorest country in the Middle East and North Africa [19]. A civil conflict that started in 2014 has resulted in large-scale human displacement, economic deterioration, significant damage to the health system, and repeated infectious disease outbreaks like cholera [20,21]. This has a significant impact on young children and infants who are vulnerable to growth failure [22,23,24,25]. Nonetheless, recent data on anthropometric failure among <6 m infants in Yemen hardly exist. While a 2023 UNICEF survey revealed a high burden of conventional failures for this age group [26], existing reports often overlook the overlapping nature of these failures. Consequently, they fail to provide a comprehensive picture of the prevalence and severity that accounts for both single and combined anthropometric failures. This study aims to bridge this gap and provide evidence of use for future nutrition policies.

The current study aims: (1) to estimate the prevalence of single, concurrent and severe forms of anthropometric failure in infants under six months attending routine health visits in two governorates in Yemen and (2) to determine which anthropometric indicator shows greater overlap with different forms of anthropometric failure.

## 2. Materials and Methods

### 2.1. Study Sites

Aden and Lahj, two neighboring Yemeni governorates with free movement between them, were selected for the study because of the high number of displaced families and a relative stable security situation [22]. Within these two governorates, four districts were randomly selected: Al-Sheikh Othman and Al-Boraiqa in Aden, and Tuban and Toor Al-Baha in Lahj lowland (LL). In each, the health facility (HF) hosting the largest vaccination center was chosen; this included: Al-Boraiqa and Al-Sheikh Othman health centers (HCs) in Aden, and Toor Al-Baha HC and Al-Wahat district hospital in Lahj. A fifth facility, Al-Habylain interdistrict hospital, was purposefully selected upon recommendation from the health authorities as it serves a large Lahj highland population across three districts. Overall, these facilities serve catchment populations ranging from 12,407 to 58,319.

### 2.2. Study Design and Sample Size

The sample size of this multi-center, facility-based cross-sectional survey was calculated using a simple proportion formula https://epitools.ausvet.com.au/oneproportion (accessed on 11 May 2022). In the absence of recent data on anthropometric failures encompassing composite failure for infants < 6 m in Yemen, a conservative estimate of 50% was adopted during planning to maximize variance and ensure a sufficiently powered sample size [27]. With 3% precision and a 95% confidence, a sample size of 1068 infants was calculated. With the addition of 10% for incomplete data, the minimum sample size required was 1175 infants. All infants < 6 m visiting the selected healthcare facilities were screened.

### 2.3. Data and Measurements

Two trained research assistants (RAs) were allocated per facility. The RAs were experienced nurses working in the nutrition department of the respective HF. All RAs received a two-day training on obtaining informed consent, anthropometric measurements and Z score calculation. During implementation, we collected data through interviews done with the primary caregiver/mother (from now on will be referred to as caregiver) using a standardized form. The data collected included basic-demographic, socio-economic and anthropometric data. Feeding practices and other risk factors are not within the scope of this paper and will be addressed in a separate analysis. We classified the household socio-economic status (SES) as low, medium and high using Fahmy and El-Sherbini’s validated tool [28]. At the beginning of enrollment, full data was collected from each infant. However, after an initial period, data collection was reduced to core demographic and anthropometric data to mitigate maternal interview fatigue in high-volume clinical settings. The RAs weighed undressed infants using a daily standardized digital baby scale EBSA-20 with 5 g precision (Zhongshan Jinli Electronic Weighing Equipment Co., Ltd., Zhongshan, China). Infant length was measured to the nearest 0.1 cm, using identical UNICEF portable wooden length-measuring boards. MUAC measurements followed standard methodology [29] using UNICEF non-stretch tape and were recorded to the nearest 0.1 cm. Measurements were taken independently by two RAs, and the average was recorded according to international standards. A threshold difference of 100 g for weight and 5 mm for length/MUAC was set for discrepancy, otherwise the measurement was repeated.

### 2.4. Data Handling and Analysis

Data were entered and analyzed using Statistical Package for Social Sciences (SPSS) version 25. Infants with missing age, weight, length or having out-of-range values (age above 6 months, length below 45 cm) were excluded from the final analysis. We used the date of birth recorded on the vaccination card or reported by the caregiver. Age in months and WAZ, LAZ and WLZ scores were calculated using WHO Anthro software version 3.2.2. Data cleaning criteria followed WHO 2006 recommendations [30]. Biologically implausible anthropometric values (WLZ: <−5 or >5; LAZ: <−6 or >6; WAZ: <−6 or >5) were flagged and removed after consistency was checked across other indicators [31].

The analysis plan partially followed the methodology previously reported by Grijalva-Eternod et al. [32]. Wasted, stunted and underweight were defined as having WLZ, LAZ or WAZ −2 z-scores, and severe wasting, stunting and underweight as −3 z scores below WHO 2006 median growth standards.

The CIAF was defined as all infants having at least one form of undernutrition (wasting, stunting or underweight). Following established protocols [6,7], six mutually exclusive categories of CIAF were defined: (1) wasting only; (2) wasting and underweight; (3) wasting, underweight and stunted; (4) stunted only; (5) stunted and underweight; (6) underweight only. Similarly, the composite index of severe anthropometric failure (CISAF) was defined as all infants having at least one form of severe anthropometry (WLZ; LAZ or WAZ < −3) using categories comparable to those of the CIAF.

To identify infants with low MUAC, three cut offs were evaluated: <10.5 cm, <11 cm, and <11.5 cm. One or more of these thresholds have been utilized in recent peer-reviewed publications evaluating nutritional status in infants < 6 m, including those below 6 weeks of age [18,32,33].

The demographic, household and anthropometric data were summarized using summary statistics. The analysis accounted for the complex survey design by adjusting for clustering at the district level. We estimated prevalence by constructing 95% confidence intervals using SPSS complex samples. The Chi-square test was applied to compare prevalence between age groups using a 3-month cut off to ensure comparability with previous studies [34,35], and a *p*-value < 0.05 was considered significant. Finally, we examined the overlap between infants having different forms of anthropometric failure (wasting, stunting, underweight, CIAF and CISAF) with the three low MUAC cut offs aforementioned and WAZ < −2.

### 2.5. Ethical Considerations

The study received approval by the institutional review board, Faculty of Medicine, University of Aden (REC-139-2022). We followed the international standards of good clinical practice, and obtained informed consent (signed or fingerprinted by the infant’s caregiver) after proper explanation in the local language. Infants identified as severely wasted or requiring medical attention were referred to the appropriate service according to the local context.

## 3. Results

### 3.1. Main Findings

#### 3.1.1. Flow of Participants

**Figure 1:**
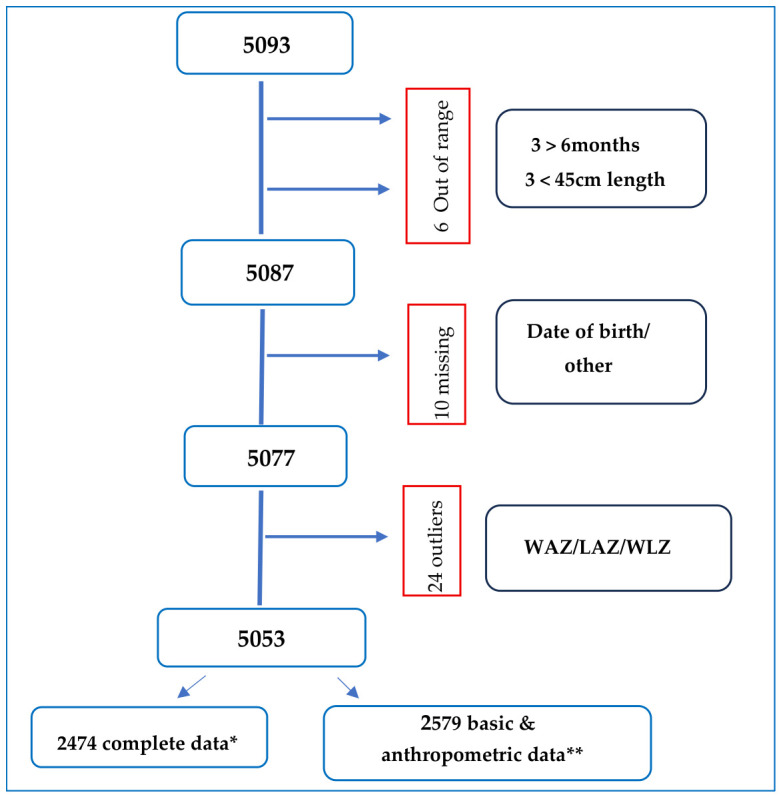
Participant flow. * Complete data included: demographic, household and anthropometric data. ** Basic data included demographic and anthropometric data only.

From December 2023 to December 2024, 5093 caregivers agreed to participate in the study. Twenty-four infants were excluded for being biologically implausible, and 5053 were included in the final analysis. In total, 2474 had complete data and 2579 had basic and anthropometric data (Figure 1). Within the latter group, 12 infants missing MUAC data were retained as they contributed to our primary conventional anthropometry and CIAF analysis.

The exclusions (WAZ < −6: N*n* = 1; LAZ < −6: *n* = 4; LAZ > 6: *n* = 4; WLZ < −5: *n* = 14; WLZ > 5: *n* = 1) made less than 0.5% of the total sample. A descriptive analysis of the characteristics of the excluded group against the study cohort is shown in Table S1. The exclusions were distributed in different age categories and zones. Overall, the excluded infant did not show systematic variation from the study cohort that would suggest selection bias.

#### 3.1.2. Sample Demographic and Anthropometric Characteristics

Infants’ mean age was 2.2 months, and fifty percent were male. The majority (66%) came from rural areas, and 22.7% were reported by their caregivers to be of small size at birth. The SES was split between medium SES 51%, low 48%, and 1% high status. More demographic and household characteristics can be found in the Supplementary Material (Table S1). Mean values of all conventional anthropometric indices, WLZ, LAZ and WAZ, were below the WHO reference median. Overall, wasting, stunting and underweight were 19.3%, 17.7% and 30.5% respectively (Table 1).

#### 3.1.3. Prevalence of Different Anthropometric Failures Including Composite Indices by Age

**Figure 2:**
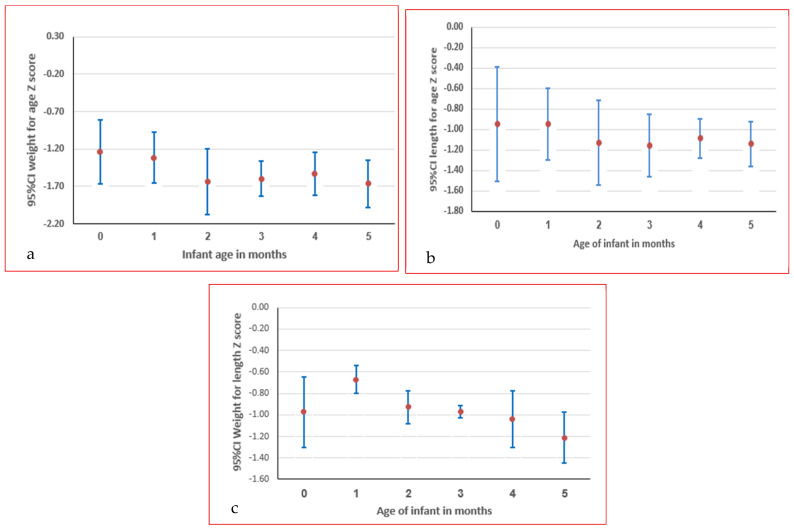
Distribution of mean anthropometric indices by age. (**a**) Mean weight for age z score; (**b**) mean length for age z score; (**c**) mean weight for length z score.

Table 2 shows the proportion of different types of conventional indicators along with CIAF and CISAF stratified by age. Overall, CIAF and CISAF comprised a significant proportion of infants, 41% and 12%, respectively. The percentage of wasting and underweight (including their severe forms) as well as CIAF and CISAF showed an increase with age. Figure 2a–c show the distribution of mean WAZ, LAZ and WLZ across different age categories.

#### 3.1.4. Prevalence of Single and Concurrent Forms of Anthropometric Failure

Table 3 presents the prevalence of single and concurrent forms of anthropometric failure in infants under 3 months (<3 m) compared to 3 months and above (≥3 m). The combined prevalence of all single failures (17.7%) was less than the combined prevalence of all concurrent failures (23.3%). The category ‘wasted and underweight’ was significantly higher among infants ≥ 3 m than among the younger infants. Conversely, the category ‘underweight only’ was significantly more prevalent among the younger age group compared to the older group. Analyses for the CISAF categories are shown in Table S2**,** highlighting that two categories were significantly more prevalent among ≥3 m infants.

#### 3.1.5. Prevalence of Low MUAC

Table 4 shows that prevalence of low MUAC was influenced by both age and the cut off applied. Across all cut offs applied, the proportion of infants with low MUAC was at its maximum at 0 months, then decreased significantly from 0 month to 1 month.

Similar patterns were observed for the prevalence of low MUAC when infants were stratified by CIAF status and caregiver-reported birth size. Regardless of their classification, all MUAC cut offs identified the highest proportion of infants at 0 months; prevalence decreased sharply thereafter (Tables S3 and S4).

#### 3.1.6. Which Indicator Shows Greater Overlap with Different Anthropometric Failures?

Table 5 shows the proportion of different anthropometric failures identified by MUAC and WAZ cut offs when applied as individual initial assessment tools. The MUAC threshold < 11.5 cm identified the highest proportion of all anthropometric failures. This captured between 28.8 and 47% of infants in the different groups of failures, and 11.5% of infants with no failure. However, WAZ < −2 showed greater overlap than MUAC cut offs; it captured between 71.6 and 92% of infants with different failures, and 0% of infants with no anthropometric failure. MUAC and WAZ demonstrated a similar pattern of overlap with CIAF categories (Table S5).

To ensure infants reported as small at birth (SAB) did not drive the superior overlap of WAZ compared to MUAC, a sensitivity analysis was conducted excluding these individuals (Table S6). Although removing SAB infants increased MUAC’s overlap with various growth failures, it also resulted in MUAC misclassifying up to 33.3% of healthy infants (No CIAF) as having growth failure. Notably, WAZ maintained greater overlap than MUAC (capturing 100% of infants with CIAF) while demonstrating a 0% misclassification rate.

## 4. Discussion

This study shows that anthropometric failure is prevalent among <6 m infants attending routine health visits in two Yemeni governorates with wasting, stunting and underweight prevalences standing at 19.3%, 17.7% and 30.5% respectively. We are the first to report CIAF among < 6 m infants in Yemen and found a worryingly high rate of 41%. Overall, 23.3% of the sample had concurrent failures, and 12% had at least one severe anthropometric failure (CISAF). When comparing <3 m and ≥3 m infants, certain forms of concurrent and severe anthropometric failure were more prevalent in ≥3 m infants. The extent to which low MUAC overlapped with and captured infants with other anthropometric failures (defined by CIAF and reported size at birth) was highly influenced by age. Our data showed that WAZ < −2 overlapped significantly more than MUAC with anthropometric failures—as defined by conventional indicators, CIAF and CISAF—in <6 m infants.

Consistent with the literature, our wasting and underweight prevalence rates were similar to those reported in a 2023 survey (19.3% vs. 17.8% and 30.5% vs. 31.9%, respectively) [26]. In contrast, our prevalence of stunting (17.7%) and severe wasting (4.2%) were lower than the national estimates reported in the same survey. This discrepancy may reflect geographic variation (our sample was drawn from governorates with low under-5 years stunting rates [36]), younger age distribution (60% of our sample was under 3 months, before stunting typically manifests [37]), and difference in health status between facility-based and community samples [38]. Overall, the high prevalence of anthropometric failures may reflect low household SES and maternal education [39]; a high prevalence of low birth weight, proxied by reported small size at birth 22.7% (this proxy is a practical LBW estimate in some low-resource settings [40]); suboptimal infant feeding practices [36] and infections [20,41]. Our finding that some concurrent and severe anthropometric failures—namely, severe wasting only, wasting and underweight, and severe wasting with severe underweight—are more prevalent in older infants is consistent with the literature [35]. In a high undernutrition-burden context such as Yemen, this pattern likely reflects the compounded effects of early growth faltering, recurrent infections, and inadequate feeding.

The study observed a high prevalence of low MUAC among infants aged 0 months. While this pattern is partially attributable to birth weight confounding—for MUAC 10.5 cm, prevalence decreased from 52.4% to 19.6% when stratified by reported birth size—a sharp decline from 0 to 1 month persisted across all sub-analyses. Furthermore, our age-stratified analysis (Table S3) revealed that the MUAC < 11.5 cm threshold yielded a prohibitively high misclassification rate, flagging 63.7% of infants with (No CIAF) at 0 months. Similar trends have been documented in other countries, such as Ethiopia [32]. This rapid decline likely reflects neonatal physiological subcutaneous fat expansion and inherent measurement imprecision in neonates [42], and not necessarily wasting alone. These findings raise the question of which MUAC cut off is appropriate for neonates and highlight the limitations of applying fixed MUAC thresholds to this vulnerable population.

In this cohort, WAZ < −2 captured a greater proportion of infants with different anthropometric failures than low MUAC. While consistent with the literature [43], our finding should be interpreted within the Yemeni context, where the high prevalence of underweight may have amplified the performance of WAZ at the expense of MUAC. In our study, this apparent advantage of WAZ < −2 refers strictly to its greater overlap with the anthropometric failures examined (including CIAF and CISAF) in this facility-based cohort and not its prognostic superiority for predicting mortality or morbidity.

Management of malnutrition in <6 m Yemeni infants focuses primarily on the inpatient treatment of severe wasting. Our findings highlight a high burden of severe anthropometric failure—as defined by the CISAF—and high burden of concurrent failures, which carry documented mortality risks [8,44,45]. These high-risk failures occur at two to four times the frequency of severe wasting reported nationally, 6.3% [26]. This suggests that current care may overlook the majority of nutritionally high-risk infants. Therefore, a key policy implication is the expansion of clinical care to prioritize the admission of infants with severe or concurrent failures, while simultaneously strengthening preventive services—specifically exclusive breastfeeding and adequate antenatal care [46,47].

The second implication of our findings is the replacement of WLZ with WAZ for the routine facility-based assessment of infants < 6 m in Yemen. Currently, Yemen’s growth monitoring program relies on WLZ. WLZ is less reliable and practical than WAZ due to length-measurement errors and higher staffing demands [12,43,48]. WAZ eliminates these logistical constraints and remains feasible when caregivers accurately report birth dates. In this cohort, WAZ < −2 overlapped with at least 72% of all conventional anthropometric failures and 100% of concurrent failures, and did not misclassify any infants free of growth failure (No CIAF). These higher-overlap and lower-misclassification advantages of WAZ were maintained when infants reported as small at birth were excluded from the analysis. This prevents overestimation of nutritionally at-risk infants and supports the programmatic utility of WAZ. Electronic scales are already available in primary healthcare centers, so transitioning to WAZ requires only minimal staff training on WAZ reference tables.

Finally, our sample was drawn from the capital and an adjacent governorate where maternal literacy and vaccination cards optimized age verification and WAZ calculation. Further multi-site, facility-based research is warranted in more remote governorates to confirm the national burden and ascertain age reporting reliability in more rural contexts. Our finding combined with this future research will help define the optimum roles of WAZ and MUAC in Yemen nationwide.

### Strengths and Limitations

This is the first study assessing undernutrition prevalence and severity among Yemeni infants < 6 m of age. A key strength is the rural representation of this sample (66%), which mirrors Yemen’s population distribution (70%). Additionally, the substantial proportion of infants reported as small at birth—reflecting the high national prevalence of low birth weight [26,49]—allowed us to evaluate MUAC and WAZ across a spectrum of vulnerability by comparing their performance in infants perceived as small versus those of normal size at birth.

Our study also has limitations. The selection of study facilities was constrained by logistical, practical, and safety considerations. While the design and analysis accounted for clustering to minimize bias, the facility-based nature of the study and its limitation to two governorates may limit the generalizability of our findings to all of Yemen. Furthermore, adjusting for the design effect resulted in wider confidence intervals, which provides an accurate reflection of the uncertainty within our estimates. Finally, the absence of gestational age data necessitated the use of chronological rather than corrected age. This may have led to the misclassification of preterm or small-for-gestational-age infants, and potentially overestimation of growth failure—particularly among infants perceived as small at birth. However, this overestimation is mitigated by the clinical utility of WAZ. Given that WAZ misclassified 0% of infants with no anthropometric failure (No CIAF), the impact of this overestimation in this fragile context would result in well-merited clinical attention and monitoring for a vulnerable segment of the infant population.

## 5. Conclusions

While conventional nutritional deficits are highly prevalent among Yemeni infants < 6 m, the total burden of anthropometric failure is significantly higher. The data revealed a critically high burden of both total severe and concurrent anthropometric failures. Our findings suggest that WAZ < −2 better captures nutritional failure in <6 m infants. Policymakers should consider the standardization of WAZ use in routine care to identify high-risk infants. Additionally, future multi-site facility-based research is needed to ascertain age reporting reliability and define optimum roles of WAZ and MUAC in remote governorates.

## Figures and Tables

**Table 1:** Anthropometric characteristics.

Infant Anthropometry (*n* = 5053)	Mean ± S.D.	Total/Global Failure * <−2 Z % (95% CI)	Severe Failure * <−3 Z (95% CI)
Weight (kg)	4.8 ± 1.10		
Length (cm)	57.23 ± 4.32		
WLZ score	−0.91 ± 1.22	19.3 (15.1; 24.3)	4.2 (2.9; 6.1)
LAZ score	−1.07 ± 1.10	17.7 (13.8; 22.5)	3.9 (2.7; 5.6)
WAZ score	−1.50 ± 1.11	30.5 (21.8; 40.7)	9.1 (7.8; 10.5)
MUAC (cm) (*n* = 5041)	12.37 ± 1.13		

* Prevalence is presented as percentage (%) of infants falling below the specified thresholds (<−2 and <−3 Z scores). According to WHO, <−2 Z denotes total (moderate and severe) anthropometric failure and <−3 Z denotes severe anthropometric failure. 95% Confidence intervals (CIs) were calculated using complex samples module to adjust for multi-stage cluster sampling design.

**Table 2:** Proportion of <6 m with different forms of anthropometric failure by age.

Anthropometric Indicator *n* = 5053	0 m (*n* = 491) (95% CI)	1 m (*n* = 1345) (95% CI)	2 m (*n* = 1180) (95% CI)	3 m (*n* = 896) (95% CI)	4 m (*n* = 678) (95% CI)	5 m (*n* = 463) (95% CI)
Wasted	20% (12.9; 29.5)	13.1% (10.7; 16.0)	17.2% (11.5; 24.9)	20.4% (14.1; 28.6)	26% (19.9; 33.0)	29.8% (24.8; 35.3)
Severely wasted	2.4% (1.5; 4.0)	2% (1.1; 3.7)	3.1% (1.7; 5.5)	5.5% (3.6; 8.2)	5.5% (2.7; 10.7)	11.4% (9.2; 14.1)
Stunted	21% (7.2; 47.6)	17.9% (11.7; 26.4)	18.9% (14.1; 24.8)	16.2% (11.3; 22.7)	15.3% (6.5; 32.1)	17.3% (7.0; 36.6)
Severely stunted	6.3 (2.5; 15.3)	3.3% (2.3; 4.6)	3.1% (1.8; 5.4)	4.6% (2.3; 8.8)	4% (1.6; 9.7)	3.2% (0.7; 13.9)
Underweight	20.2% (9.0; 39.1)	26.3% (15.2; 41.6)	36.9% (22.5; 54.0)	30.8% (24.8; 37.5)	30.5% (24.1; 37.8)	36.3% (28.0; 45.5)
Severely underweight	7.9% (2.3; 24.1)	6.7% (3.6; 12.1)	9.7% (7.6; 12.3)	9.3% (6.7; 12.6)	9.6% (5.3; 16.7)	14.5% (8.2; 24.4)
Composite indices						
CIAF	37.7% (24.0; 53.6)	37.4% (27.5; 48.5)	45.1% (34.9; 55.7)	40.6% (35.9; 45.5)	40.9% (28.5; 54.4)	44.7% (32.8; 57.2)
CISAF	11.6% (5.0; 24.8)	9.1% (5.9; 13.8)	12.1% (9.8; 14.9)	13.3% (9.8; 17.8)	12.7% (6.3; 23.9)	17.5% (10.1; 28.7)
Composite indices of total sample						
CIAF	40.9% (34.7; 47.5)					
CISAF	12.0% (10.8; 13.4)					

95% Confidence intervals (CIs) were calculated using complex samples module to adjust for multi-stage cluster sampling design.

**Table 3:** Proportion of CIAF categories by age.

Anthropometric Indicator	All *n* = 5053 % (95% CI)	<3 m *n* = 3016 % (95% CI)	≥3 m *n* = 2037 % (95% CI)	*p*-Value
Wasted only	5.5 (2.2; 13.1)	6.0 (2.4; 14.4)	4.6 (1.8; 11.3)	0.267
Wasted and underweight	10.6 (5.4; 19.6)	7.0 (3.6; 13.0)	15.9 (9.2; 25.8)	0.011
Wasted, underweight and stunted	3.2 (2.3; 4.5)	2.8 (1.6; 4.7)	3.9 (2.1; 7.1)	0.346
Stunted only	5 (4.6; 5.5)	5 (3.9; 6.3)	5.1 (3.6; 7.1)	0.938
Stunted and underweight	9.5 (5.7; 15.5)	11.0 (6.4; 18.3)	7.2 (2.7; 17.5)	0.307
Underweight only	7.2 (3.3; 14.9)	8.6 (3.9; 17.8)	5 (2.1; 11.5)	0.045

**Table 4:** Proportion of infants with low MUAC according to different cut offs by age.

	All *n* = 5041 % (95% CI)	0 m *n* = 489 % (95% CI)	1 m *n* = 1344 % (95% CI)	2 m, *n* = 1174 % (95% CI)	3 m *n* = 895 % (95% CI)	4 m *n* = 676 % (95% CI)	5 m *n* = 463 % (95% CI)
MUAC < 10.5 cm	6 (2.4; 14.3)	33.1 (18.1; 52.6)	5.1 (2.4; 10.5)	2.1 (0.7; 6.1)	2.5 (1.0; 5.8)	1.3 (0.7; 2.7)	3.9 (1.9; 7.8)
MUAC < 11.0 cm	10.9 (4.3; 25.0)	47.9 (27.6; 68.8)	12 (6.1; 22.3)	5.4 (1.7; 15.8)	3.9 (1.7; 8.9)	2.5 (1.3; 5.0)	8.4 (5.5; 12.6)
MUAC < 11.5 cm	18.6 (7.1; 40.7)	64.4 (38.6; 83.9)	23.4 (11.4; 42.3)	11.6 (3.7; 30.9)	7.8 (3.2; 17.9)	6.8 (2.9; 15.1)	11.9 (8.1; 17.2)

**Table 5:** Overlap between low MUAC and WAZ cut offs with different forms of anthropometric failure.

*n* = 5041	Wasted *n* = 972	Stunted *n* = 893	Underweight *n* = 1534	CIAF *n* = 2062	CISAF *n* = 607	No CIAF *n* = 2979
MUAC < 10.5 cm	13.5 (7.6; 22.7)	12.7 (4.6; 30.5)	11 (4.1; 26.6)	10.3 (4.2; 23.1)	20.4 (10.1; 36.9)	3.1 (0.8; 10.9)
MUAC < 11.0 cm	22.3 (13.2; 35.3)	20.3 (7.0; 46.3)	19.1 (6.6; 44.1)	17.8 (7.0; 38.4)	33.1 (16.2; 55.9)	6.1 (1.8.; 18.8)
MUAC < 11.5 cm	36.1 (20.2; 55.8)	29.9 (10.2; 61.6)	29.7 (10.1; 61.3)	28.8 (10.9; 57.1)	47 (26.0; 69.1)	11.5 (3.4; 32.3)
WAZ < −2	71.6 (39.8; 90.6)	71.8 (63.6; 78.8)	100 (100; 100)	74.4 (58.2; 85.8)	92.1 (83.1; 96.5)	0 (0; 0)

## Data Availability

The data that support the findings of this study are not publicly available due to ethical restrictions. They will be made available from the corresponding author upon reasonable request.

## Supplementary Materials

The following supporting information can be downloaded at https://www.mdpi.com/article/10.3390/nu18142283/s1: Table S1: Sample characteristics; Table S2: Prevalence of CISAF categories by age; Table S3: Proportion of low MUAC according to CIAF status; Table S4: Proportion of low MUAC according to reported size at birth; Table S5: Overlap between low MUAC cut offs and different CIAF categories; Table S6. Overlap between low MUAC and WAZ cut offs with different forms of anthropometric failure excluding infants reported as small at birth.

## Abbreviations

The following abbreviations are used in this manuscript:

<3 m	Less than 3 months
≥3 m	Three months and above
<6 m	Under six months
CIAF	Composite index of anthropometric failure
CISAF	Composite index of severe anthropometric failure
HC	Health center
HF	Health facility
Lahj HL	Lahj lowland
Lahj LL	Lahj lowland
LAZ	Length for age z score
MUAC	Mid-upper arm circumference
SAB	Small at birth
SAM	Severe acute malnutrition
SD	Standard deviation
SES	Socio-economic status
UNICEF	United Nations Children Fund
WHO	World Health Organization
WAZ	Weight for age z score
WLZ	Weight for length z score
