# Supplementary material for: Undernutrition in Infants Aged Under Six Months: A Multi-Center Cross-Sectional Study in Two Governorates in Yemen
Source: Nutrients. 2026 Jul 12;18(14):2283. doi: 10.3390/nu18142283 (PMC13415199; doi:10.3390/nu18142283)
Supplement: Supplementary file 1 [file nutrients-18-02283-s001.zip › nutrients-4307056-supplementary.pdf]

Undernutrition in Infants Aged under Six Months: A Multi-Centre Cross-Sectional Study in Two Governorates in Yemen (supplementary material)

Table S1. Sample characteristics

	Study cohort (n= 5053)		Excluded group (n=24)	
	**Mean (S.D) or Freq**	**(%)***	**Mean (S.D) or Freq**	**(%)***
Infant age (months)	2.26 (1.45)		2.42 (1.28)	
Gender				
Male	2539	(50.2)	17	(70.8)
Female	2514	(49.8)	7	(29.2)
Place of residence				
Urban	1712	(33.9)	5	(20.8)
Rural	3341	(66.1)	19	(79.2)
Type of residence				
Permanent	4927	(97.5)	23	(95.8)
Displaced/migrant	126	(2.5)	1	(4.2)
Zone of recruitment				
Aden	1849	(36.6)	5	(20.8)
Lahj LL	1830	(36.2)	11	(45.9)
Lahj HL	1374	(27.2)	8	(33.3)
Household Characteristics	**Study cohort n=2474**		**Excluded group n=21**	
	**Median (Iqr) or Frq**	**(%)***	**Median (Iqr) or Frq**	**(%)***
Mother education				
No education:	420	(17)	5	(23.8%)
Below secondary:	980	(39.6)	9	(42.9%)
Secondary:	807	(32.6)	4	(19%)
University and above:	267	(10.8)	3	(14.3%)
Father education				
No education:	188	(7.6)	2	(9.5%)
Below secondary:	564	(22.8)	6	(28.5%)
Secondary:	1163	(47)	9	(42.9%)
University and above:	559	(22.6)	4	(19%)
Maternal work				
Yes:	135	(5.5)	1	(4.8%)
No:	2339	(94.5)	20	(95.2%)
Father work				
Yes:	2387	(96.5)	20	(95.2 %)
No:	87	(3.5)	1	(4.8%)
Reported size at birth				
Small (<2500 gm)	562	22.7	9	42.9
Medium (2500-4000 gm)	1887	76.3	12	57.1
Large	25	1	0	0
# of family members				
	6 (4;8)		1 (0;5)	
Socio-economic status				
Low:	1189	(48)	2	(9.5%)
Medium:	1262	(51)	16	(76.2%)
High:	23	(1)	3	(14.3%)

*Percentages are calculated based on the available data; the denominators vary across sections due to missing data. Complete demographic and anthropometric records were available for the full study cohort (n = 5,053) and all excluded cases (n = 24). Household and socio-economic characteristics were only available for (n = 2,474) of the study cohort and (n = 21) of the excluded group.

Undernutrition in Infants Aged under Six Months: A Multi-Centre Cross-Sectional Study in Two Governorates in Yemen (supplementary material)

Table S2. Prevalence of CISAF categories by age

Anthropometric indicator	All n=5053 % (95% CI	< 3m n= 3016 % (95% CI)	≥ 3m n=2037 % (95% CI)	p-value
CISAF	12 (10.8;13.4)	10.7 (7.8;14.5)	14 (9.4;20.5)	.311
Severe wasted only	1.6 (.8;3.2)	1.3 (.7;2.5)	2 (.8;4.6)	.020
Severe wasted and severe underweight	2.4 (1.2;4.8)	1 (.5;2.3)	4.5 (3.1;6.5)	.001
Severe wasted, severe underweight and severe stunted	.2 (.1; .5)	.2 (.1;.5)	.3 (.1;1.0)	.237
Severe stunted only	1.4 (.9;2.3)	1.3 (.8;2.3)	1.5 (.6;3.8)	.749
Severe stunted and severe underweight	2.2 (1.2;4.0)	2.2 (1.5;3.3)	2.2 (0.7;7.1)	.990
Severe underweight only	4.2 (2.9;6.0)	4.6 (2.2;9.6)	3.5 (2.1;5.8)	.535

Table S3. Proportion of low MUAC according to CIAF status

With CIAF n=2062	0m n=185 % (95% CI)	1m n=502 (95% CI)	2m n= 529 (95% CI)	3m n=363 (95% CI)	4m n=276 % (95% CI)	5m n= 207 % (95% CI)
MUAC <10.5 cm	47 (16.9;79.5)	10.7 (3.7;27.3)	4.5 (1.2;15.4)	6.0 (2.2;15.6)	3.2 (1.3;8.1)	8.2 (3.5;18.2)
MUAC <11.0 cm	55.7 (18.1;87.7)	23.5 (9;48.8)	10.9 (2.7;34.8)	9.3 (3.6;22.2)	5.8 (2.2;14.2)	18.4 (10.7;29.7)
MUAC <11.5 cm	64.9 (19.8;93.2)	39 (13.3;72.6)	21.4 (5.7;55.2)	17.9 (7.3;37.4)	16.2 (8.8;28.2)	26.1 (18.4;35.6)
WAZ <-2	53.5 (33.5;72.4)	70.4 (50.9;84.5)	81.8 (58.5;93.4)	75.8 (59.2;87.1)	74.7 (59.5;85.6)	81.2 (77.9;84.1)
**No CIAF n=2979**	**0m n=304 % (95% CI)**	**1m n=842 % (95% CI)**	**2m n=645 % (95% CI)**	**3m n=532 % (95% CI)**	**4m n=400 % (95% CI)**	**5m n= 256 % (95% CI)**
MUAC <10.5 cm	24.5 (10.4;47.5)	1.7 (0.9;3)	0.2 (0.0;1.9)	0 (;0)	0 (0;0)	0.4 (0;6.9)
MUAC <11.0 cm	42.8 (22.9;65.3)	5.1 (2.3;10.9)	.8 (0.1;4.2)	.2 (0;2.7)	.2 (0;4.2)	0.4 (0;6.9)
MUAC <11.5 cm	63.7 (40.8;81.7)	14.1 (7.2;26)	3.4 (.4;21.9)	.9 (.1;8.1)	.2 (0;4.2)	0.4 (0;6.9)
WAZ <-2	-----------	------------	-------------	------------	-----------	-----------

Undernutrition in Infants Aged under Six Months: A Multi-Centre Cross-Sectional Study in Two Governorates in Yemen (supplementary material)

Table S4. Proportion of low MUAC according to reported size at birth

Small at birth n=562	0m n=42 % (95% CI)	1m n=170 (95% CI)	2m n= 122 (95% CI)	3m n=88 (95% CI)	4m n=91 % (95% CI)	5m n= 49 % (95% CI)
MUAC <10.5 cm	52.4 (20.9;82)	14.1 (7.9;23.9)	10.7 (3.3;29.3)	8.0 (1.2;37.3)	8.8 (2.1;30)	18.4 (3.6;57.9)
MUAC <11.0 cm	64.3 (32.5;87.1)	24.7 (19.1;31.4)	17.2 (8.2; 32.5)	12.5 (3.9;33.6)	13.2 (4.9;30.9)	28.6 (9.4;60.6)
MUAC <11.5 cm	76.2 (51.6;90.6)	41.2 (37.1;45.3)	31.1 (20.8;43.8)	26.1 (10;53)	27.5 (22;33.7)	34.7 (12.2;67)
WAZ <-2	31 (8.8;67.6)	32.4 (19.7;48.2)	45.9 (27.8;65.2)	38.6 (27.9;50.5)	54.9 (38.2;70.6)	67.3 (61.8;72.4)
**Not small at birth n= 1912**	**0m n=102 % (95% CI)**	**1m n=538 (95% CI)**	**2m n=467 (95% CI)**	**3m n=341 (95% CI)**	**4m n=274 % (95% CI)**	**5m n= 190 % (95% CI)**
MUAC <10.5 cm	19.6 (8;40.6)	4.6 (2.5;8.5)	1.7 (0.5;6.2)	3.8 (1.5;9.6)	0.4 (0;3.3)	4.2 (1;16)
MUAC <11.0 cm	34.3 (14.5;61.7)	11.9 (6.4;20.9)	5.4 (1.8;15.1)	6.5 (3;13.3)	1.8 (0.3;9.6)	12.6 (5.7;25.5)
MUAC <11.5 cm	55.9 (29.7;79.1)	23.2 (11.9;40.3)	13.7 (5;32.2)	11.4 (7.4;17.2)	5.5 (3.8;7.9)	17.4 (11;26.3)
WAZ <-2	7.8 (1.7;29	18.4 (9.4;32.8)	27.8 (12.1;52)	28.2 (17.8;41.4)	28.5 (24.3;33)	40.5 (31.6;50.1)

Table S5. Overlap between low MUAC cut offs and different CIAF categories

MUAC threshold n=2062	Wasted only n=276	Wasted & underweight n=532	Wasted, stunted & underweight n=164	Stunted and underweight n=477	Stunted only n=252	Underweight only n=361
MUAC <10.5 cm	10.9 (4.6;23.5)	10 (5.2;18.2)	29.3 (16.7;46.0)	10.7 (2.8;33.4)	5.6 (1.4;19.6)	4.7 (.6;29.3)
MUAC <11.0 cm	18.1% (10.8;28.8)	18 (9.8;30.8)	43.3 (23.4;65.6)	18 (4.7;49.6)	9.5 (2.3;32.5)	11.1 (1.9;44.7)
MUAC <11.5 cm	35.9 (25.5;47.8)	29.5 (14.6;50.6)	57.9 (35.2;77.7)	27.9 (6.7;67.7)	15.5 (4.2;43.3)	19.7 (3.3;63.6)
WAZ <-2	0 (0;0)	100 (100;100)	100 (100;100)	100 (100;100)	0 (0;0)	100 (100;100)

Table S6. Overlap between low MUAC and WAZ cut offs with different forms of anthropometric failure excluding infants reported as small at birth

n=1912	Wasted n=350	Stunted n=294	Underweigh n=488	CIAF n=714	CISAF n=227	No CIAF n=1198
MUAC <10.5 cm	45.3 (18.4;75.3)	34.7 (28.4;41.5)	66.7 (37.4;87.0)	73.3 (49.0;88.7)	48.0 (20.3;77.0)	26.7 (11.3;51.0)
MUAC <11.0 cm	45.7 (20.9;72.9)	29.7 (18.4;44.2)	60.0 (34.4;81.1)	70.9 (47.9;86.5)	42.3 (15.4;74.7)	29.1 (13.5.;52.1)
MUAC <11.5 cm	42.0 (23.5;63.2)	25.2 (16.5;36.5)	61.4 (35.9;81.9)	66.7 (48.5;86.5)	31.2 (11.5;61.4)	33.3 (19.0;51.5)
WAZ <-2	53.5 (32.0;61.6)	39.1 (30.2;48.9)	100 (100:100)	100.0 (100.;100.0)	42.0 (31.4;53.4)	0 (0;0)
